# Understanding and defining sanitation insecurity: women’s gendered experiences of urination, defecation and menstruation in rural Odisha, India

**DOI:** 10.1136/bmjgh-2017-000414

**Published:** 2017-10-09

**Authors:** Bethany A Caruso, Thomas F Clasen, Craig Hadley, Kathryn M Yount, Regine Haardörfer, Manaswini Rout, Munmun Dasmohapatra, Hannah LF Cooper

**Affiliations:** 1 Department of Behavioral Sciences and Health Education, Rollins School of Public Health, Emory University, Atlanta, Georgia, USA; 2 Department of Environmental Health, Rollins School of Public Health, Emory University, Atlanta, Georgia, USA; 3 Department of Anthropology, Emory University, Atlanta, Georgia, USA; 4 Hubert Department of Global Health, Rollins School of Public Health, Emory University, Atlanta, Georgia, USA; 5 Department of Sociology, Emory University, Atlanta, Georgia, USA; 6 London School of Hygiene and Tropical Medicine, London, UK

**Keywords:** qualitative study, environmental health, hygiene, public health

## Abstract

**Background:**

Research suggests that the lived experience of inadequate sanitation may contribute to poor health outcomes above and beyond pathogen exposure, particularly among women. The goal of this research was to understand women’s lived experiences of sanitation by documenting their urination-related, defecation-related and menstruation-related concerns, to use findings to develop a definition of *sanitation insecurity* among women in low-income settings and to develop a conceptual model to explain the factors that contribute to their experiences, including potential behavioural and health consequences.

**Methods:**

We conducted 69 Free-List Interviews and eight focus group discussions in a rural population in Odisha, India to identify women’s sanitation concerns and to build an understanding of sanitation insecurity.

**Findings:**

We found that women at different life stages in rural Odisha, India have a multitude of unaddressed urination, defecation and menstruation concerns. Concerns fell into four domains: the sociocultural context, the physical environment, the social environment and personal constraints. These varied by season, time of day, life stage and toilet ownership, and were linked with an array of adaptations (ie, suppression, withholding food and water) and consequences (ie, scolding, shame, fear). Our derived definition and conceptual model of sanitation insecurity reflect these four domains.

**Discussion:**

To sincerely address women’s sanitation needs, our findings indicate that more is needed than facilities that change the physical environment alone. Efforts to enable urinating, defecating and managing menstruation independently, comfortably, safely, hygienically, privately, healthily, with dignity and as needed require transformative approaches that also address the gendered, sociocultural and social environments that impact women despite facility access. This research lays the groundwork for future sanitation studies to validate or refine the proposed definition and to assess women’s sanitation insecurity, even among those who have latrines, to determine what may be needed to improve women’s sanitation circumstances.

**Key questions**

**What is already known about this topic?**

- Poor or non-existent sanitation facilitates faecal pathogen exposure, which can lead to numerous infectious disease outcomes, including diarrhoea, soil-transmitted helminth infection, trachoma and schistosomiasis among others.
- A small but growing line of research suggests that inadequate sanitation poses specific health risks to women beyond infectious diseases, like adverse pregnancy outcomes, non-partner violence and stress.

**What are the new findings?**

- Toilet access enables a choice but not a solution; women identified a multitude of concerns associated with their urination, defecation and menstruation experiences even if they owned functional toilets.
- Women’s ability to attend to their needs were strained by the gendered roles they were expected to uphold and their access to and dependence on social support systems.
- Life stage, weather conditions and time of day influenced the intensity of women’s sanitation-related concerns.

**Recommendations for policy**

- Using our findings, we propose a definition for *sanitation insecurity*, which can initiate discourse and further research to validate or refine the definition.
- Current sanitation global policy and practice typically focuses on defecation, leaving women’s urination and menstruation-related needs underserved.
- Sanitation programmes have the potential to be gender transformative through programming that tackles the social norms that make women’s sanitation needs second to the obligations she has to her household and family.

## Introduction

Globally, approximately 2.3 billion people lack access to basic sanitation, unshared household facilities that hygienically separate human excreta from human contact.^1^ Of these, 892 million people lack access to any sanitation and practice open defecation.^1^ Poor or non-existent sanitation facilitates faecal pathogen exposure, which is associated with multiple infectious disease outcomes, including diarrhoea, soil-transmitted helminth infection, trachoma and schistosomiasis.^2 3^ The Millennium Development Goals aimed to increase coverage of improved sanitation by 2015 to combat these health impacts. Recognising that coverage alone is insufficient, the Sustainable Development Goals go further, calling for access to adequate and equitable sanitation and hygiene for all, with special attention to the needs of women and girls, including managing defecation, urination and menstruation needs with dignity.^4^

India represents a major sanitation challenge; 40% of the population lack sanitation access, including 56% of rural residents.^1^ A succession of government programmes has emphasised building toilets to end open defecation. The current programme, the Swachh Bharat Mission, aims to provide sanitation to all households to end open defecation by October 2019^5^. Yet, rigorous evaluations of the previous campaign found limited increases in latrine coverage and no detectable health impacts.^6–8^ Furthermore, overall use of latrines built through these campaigns also remains low.^9–11^

A small but growing line of research suggests that inadequate sanitation poses health risks beyond infectious diseases to women in India and beyond. Open defecation has been associated with adverse pregnancy outcomes in India and higher odds of non-partner violence in India and Kenya.^12–14^ Greater access to improved sanitation has been associated with decreased odds of maternal mortality.^15^ In India and Kenya, women consider their sanitation conditions to cause stress due to compromised privacy, inability to change conditions and the potential for harm when addressing needs.^16–20^ Women in Uttar Pradesh, India reported increased challenges defecating when menstruating, noting a risk of greater shame if seen.^20^ In rural Odisha, research found that women lacked power, control of money and confidence, which men corroborated, resulting in their exclusion from decision-making, particularly regarding toilet construction.^21^ Research that further elucidates women’s sanitation experiences and needs may help explain sanitation behaviour and reveal additional health impacts.^22^

Researchers have created definitions and measures for *food* and *water insecurity* that account for individuals’ lived experiences, yet *sanitation insecurity* has yet to be defined. Definitions for both food and water insecurity are multidimensional, reflecting biological needs which may be measured objectively by assessing food and water sources or amounts ingested or used, and social needs that consider perceptions, experiences and culture.^23–25^ Food and water insecurity researchers have created measures that reflect the sociocultural context in order to assess how insecurity may be experienced.^23 26 27^ O’Reilly has conceptualised *toilet insecurity* as ‘when safe, usable toilets are not available’ (p. 19).^28^ While safety and usability of a facility are critical, we expect women may experience sanitation insecurity regardless of having a safe and functional toilet. As with research on water and food insecurity, we hypothesise that *sanitation insecurity* extends beyond access, is multidimensional, and also considers experiences, perceptions and preferences, associated with the sociocultural context.

This research aimed to: (1) document the full range of voiced urination, defecation and menstruation concerns of women in rural Odisha, India, and to use findings to (2) develop a definition and (3) conceptual model of *sanitation insecurity* that shows the factors that contribute to sanitation insecurity and the impacts it may have on behaviour and health. We focus on urination, defecation and menstrual hygiene, as these are the key personal behaviours noted in goal 6 of the Sustainable Development Goals. We document concerns because concerns may be ever-present, impacting behaviour, events and life, regardless of whether or not they are actualised. A woman may worry about assault while tending to her needs, for example, but never be assaulted. Her concern for harm, however, influences her decision-making, behaviour and well-being. Her concern becomes her experience. By documenting these insights, we strive not to ignore women’s resilience or depict women solely as vulnerable. Rather, we seek to unpack their experiences to inform programmes serving women’s needs, identify drivers of latrine use and non-use and uncover potentially overlooked health risks. Our approach strives to move beyond sanitation research previously carried out in Odisha that identified environmental, social and sexual stressors contributing to sanitation-related psychosocial stress.^17 18^ We suspect that women’s concerns, their sanitation insecurity, contribute to stress, but may also influence depression, well-being and overall quality of life.

Our evidence-based definition of sanitation insecurity can eventually help establish a standard for assessing sanitation programmes. This study served as the basis of a sanitation insecurity measure developed by the authors. The measure is expected to help estimate the intensity of insecurity, inform and evaluate interventions and programmes to better suit women’s needs, and test associations with various outcomes.^29^ Specifically, we hypothesise that sanitation insecurity may be associated with mental health, like anxiety or depression, agency, economic productivity and educational outcomes, regardless of access to a functional latrine. We acknowledge that grounding this research in a specific population raises questions about applicability to other populations and settings. Nonetheless, we envision this work to be the start of a necessary discourse and that further research with other populations will lead to validation or adjustment as needed.

## Methods

### Setting and population

This research was conducted in March–April 2014 in Odisha, India within a subsample of rural communities previously engaged in a cluster randomised trial. The trial evaluated the impact of a rural sanitation intervention within the context of India’s Total Sanitation Campaign.^30^ Participants were either from a community that received the intervention or that served as controls. Toilets were typically outside the home, required fetched water for manual flushing and had cement brick walls. Not all had roofs or doors because these were not subsidised. Additional information about the setting and intervention are published elsewhere.^31^

Odisha has 33% of the population living below the poverty line, which is higher than 24 of the 30 states.^32^ Attainment of secondary education by women in Odisha is among the lowest nationally and women’s labour force participation is declining.^33^

### Data collection

Free-list interviews (FLIs) and focus group discussions (FGDs) were used to understand women’s voiced concerns and to build an understanding of sanitation insecurity.^34^ Two coauthors experienced in qualitative methods and fluent in English and Oriya (MR and MD) conducted all interviews and discussions.

#### Free-list interviews

Free-listing, an elicitation technique for understanding shared perceptions among a group of individuals,^34^ aimed to learn about women’s urination, defecation and menstruation concerns and determine the extent concerns were shared among participants.

Members of the research team purposively selected eight communities that varied along four dimensions: previous intervention status, toilet coverage, water access and seasonal conditions.

When sampling individuals, we sought variation across four life stages: (1) unmarried women (UMW) living with their parents; (2) women who had recently (past 3 years) married (RMW); (3) women married (MW) over 3 years and (4) women older than 49 years (OW). We believed sanitation insecurity would vary across these stages. UMW living in their parents’ home typically have greater resource control and thus exert control over their personal hygiene more than RMW; RWM depend on others because they have limited independent mobility outside the home.^35 36^ MW typically have greater mobility freedom and social status than RMW.^37^ OW have increased incontinence risk, unique needs related to ageing, like difficulty walking or squatting, and are under-represented in national surveys and sanitation studies.^38^ Community contacts aided recruitment.

We aimed to interview at least 64 women one-on-one (two per stage per community)—more than the 30 recommended for free lists—due to the variability in our sample.^34^ We asked women to list concerns ‘women in this community’ had when urinating, defecating and menstruating, and probed to identify temporal influence (eg, diurnal, seasonal) and variation across pregnancy and dependency status. Menopausal women answered menstruation questions based on memory. All interviews were conducted in private spaces, typically within the home, and lasted 30–90 min.

#### Focus group discussions

FGDs aimed to elicit detail about concerns reported in interviews. They enable participant discussion and served to discern if concerns reported in interviews were widely held. FGD tools were developed based on free-list interview results. We aimed to conduct eight FGDs across four communities, four with UMW and four with women married for any time period (RMW, MW, OW). We asked women to discuss urination, defecation and menstruation concerns and queried concerns mentioned in the interviews if not discussed organically. FGDs lasted 1–2 hours and were held in private spaces (school, temple or house).

### Data management and analysis

Interviews and FGDs were digitally recorded and translated directly into English. MR and MD listed all concerns reported during each interviews and listened to recordings to verify lists. BAC recreated the lists from transcripts for reliability, generated frequencies of noted concerns and created analytic codes from the concerns and applied them to both interview and FGD transcripts using MAXQDA software. We then used applied thematic analysis to examine themes, present participant’s voiced experiences and build conceptual models.^39^ Specifically, for each concern we aggregated coded text into summative tables, sorted tables by participant type to identify variation across stage and wrote memos for conceptual depth. To generate the definition, we pulled key themes from each of the dimensions identified. The definition, therefore, reflects the specific and subjective circumstances of the population engaged.

### Ethics

The Emory University Institutional Review Board (Atlanta, Georgia, USA) and KIIT University Ethics Review Committee (Bhubaneswar, India) approved study protocols. Women provided oral consent prior to participation.

## Results

### Participant characteristics

Sixty-nine women aged 18–75 years (16 UMW, 12 RMW, 22 MW, 19 OW) participated in interviews and 46 women aged 18–70 years participated in 8 FGDs (5–7 participants each; 23 unmarried and 23 married women) (table 1). For interview participants, 100% were Hindu, 26% had at least some primary education, 66% were general caste, 62% had children and 54% had a toilet within their household compound. For FGD participants, 98% were Hindu, 28% had at least some primary education, 65% were general caste, 50% had children and 59% had a toilet within their household compound. No recently married women participated in FGDs; family members did not give them permission.

**Table 1:** Demographic information for participants in FLI (n=69) and FGD (n=46)

	All		Unmarried women		Recently married women		Married women		Women older than 49 years	
FLI participants	69		16	23%	12	17%	22	32%	19	28%
Intervention community (vs control)	28	41%	5	31%	4	33%	9	41%	10	53%
Age*	36.6	(18–75)	20.7	(18–28)	23.2	(20–27)	34.0	(24–47)	61.3	(50–75)
Education										
None	16	23%	0	0%	0	0%	4	18%	12	63%
Some primary	18	26%	1	6%	3	25%	7	32%	7	37%
Some secondary	28	41%	10	63%	9	75%	9	41%	0	0%
Some tertiary	7	10%	5	31%	0	0%	2	9%	0	0%
Below poverty line card†	55	85%	14	88%	11	100%	15	75%	15	83%
Hindu	69	100%	16	100%	12	100%	22	100%	19	100%
Caste‡										
Brahmin	4	6%	1	7%	0	0%	2	9%	1	5%
General caste	44	66%	12	80%	8	73%	12	55%	12	63%
Scheduled caste (SC)	5	7%	0	0%	0	0%	3	14%	2	11%
Other backward caste (OBC)	12	18%	2	13%	3	27%	4	18%	3	16%
Scheduled tribe	2	3%	0	0%	0	0%	1	4%	1	5%
Children	43	62%	0	0%	4	33%	20	91%	19	100%
Water source within compound	43	63%	12	75%	7	58%	13	59%	11	61%
Toilet within compound	37	54%	10	63%	9	75%	9	41%	9	47%
Focus group discussion participants	46		23	50%			16	35%	7	15%
Intervention community	22	48%	10	43%			7	44%	5	71%
Age*	30.7	(18–70)	19.2	(18–23)			34.8	(20–48)	59.7	(51–70)
Education										
None	1	2%	0	0%			0	0%	1	14%
Some primary	13	28%	0	0%			8	50%	5	72%
Some secondary	12	26%	5	22%			6	38%	1	14%
Some tertiary	20	44%	18	78%			2	12%	0	0%
Below poverty line card†	29	67%	16	70%			10	71%	3	50%
Hindu	45	98%	22	96%			16	100%	7	100%
Caste‡										
Brahmin	1	2%	1	4%			0	0%	0	0%
General caste	30	65%	12	52%			11	69%	7	100%
SC	8	17%	5	22%			3	19%	0	0%
OBC	7	15%	5	22%			2	13%	0	0%
Scheduled tribe	0	0%	0	0%			0	0%	0	0%
Children	23	50%	0	0%			16	100%	7	100%
Water source within compound	32	70%	16	70%			11	69%	5	71%
Toilet within compound	27	59%	14	61%			8	50%	5	71%

*Not all women know their age; some guessed.

†Missing data for four FLI women; missing data for three FGD women.

‡ Missing data for two FLI women. While only two women reported to be from ‘scheduled tribes’, <1% of Puri residents identify as from scheduled tribes.^49^

FGD, focus group discussions; FLI, free-list interviews.

### Sanitation concerns are multidimensional

Nearly all women in interviews indicated concerns about urination (91%), defecation (94%) and menstruation (97%). They identified multiple unique concerns for each of these behaviours (urination: 29 concerns; defecation: 39 concerns; menstruation 32 concerns; see table 2 for top concerns and frequencies and online supplementary tables 1-4 for full lists and concern definitions). Women in FGDs confirmed the concerns noted in interviews.

10.1136/bmjgh-2017-000414.supp1 Supplementary file 1

**Table 2:** Type and frequency of urination, defecation and menstruation concerns overall, and by participant type and latrine status*

Concern	All		Unmarried		Recently married		Married		Older woman		Toilet		No toilet	
Urination†														
Urination place	47	74.6%	11	73.3%	10	90.9%	14	66.7%	12	75.0%	25	73.5%	22	75.9%
People	42	66.7%	14	93.3%	6	54.5%	12	57.1%	10	62.5%	22	64.7%	20	69.0%
Fear	40	63.5%	13	86.7%	10	90.9%	10	47.6%	7	43.8%	22	64.7%	17	58.6%
Need support	26	41.3%	10	66.7%	5	45.5%	7	33.3%	4	25.0%	14	41.2%	12	41.4%
Wet	21	33.3%	9	60.0%	3	27.3%	4	19.0%	5	31.3%	9	26.5%	12	41.4%
Squat	21	33.3%	0	0.0%	3	27.3%	6	28.6%	12	75.0%	13	38.2%	8	27.6%
Urine infection	19	30.2%	6	40.0%	4	36.4%	6	28.6%	3	18.8%	10	29.4%	9	31.0%
Get dirty	17	27.0%	9	60.0%	3	27.3%	4	19.0%	1	6.3%	8	23.5%	9	31.0%
Suppress	16	25.4%	5	33.3%	5	45.5%	3	14.3%	3	18.8%	11	32.4%	5	17.2%
Defecation†														
Defecation place	47	72.3%	10	71.4%	8	72.7%	15	71.4%	14	73.7%	17	51.5%	30	96.8%
Fear	36	55.4%	10	71.4%	5	45.5%	12	57.1%	9	47.4%	16	48.5%	20	64.5%
Need support	33	50.8%	5	35.7%	6	54.5%	13	61.9%	9	47.4%	16	48.5%	17	54.8%
People	27	41.5%	9	64.3%	5	45.5%	7	33.3%	6	31.6%	8	24.2%	19	61.3%
No proper facility	23	35.4%	3	21.4%	3	27.3%	10	47.6%	7	36.8%	1	3.0%	22	71.0%
Get dirty	21	32.3%	5	35.7%	5	45.5%	6	28.6%	5	26.3%	6	18.2%	15	48.4%
Support others	20	30.8%	3	21.4%	3	27.3%	4	19.0%	10	52.6%	9	27.3%	11	35.5%
Water	18	27.7%	3	21.4%	3	27.3%	7	33.3%	4	21.1%	12	36.4%	6	19.4%
Walk	17	26.2%	1	7.1%	1	9.1%	5	23.8%	9	47.4%	6	18.2%	11	35.5%
Suppress	15	23.1%	6	42.9%	2	18.2%	5	23.8%	2	10.5%	6	18.2%	9	29.0%
Dependents	14	21.5%	2	14.3%	3	27.3%	5	23.8%	4	21.1%	8	24.2%	6	19.4%
Health	13	20.0%	5	35.7%	1	9.1%	4	19.0%	3	15.8%	5	15.2%	8	25.8%
Squat	13	20.0%	0	0.0%	2	18.2%	6	28.6%	5	26.3%	8	24.2%	5	16.1%
Support barrier	13	20.0%	4	28.6%	2	18.2%	4	19.0%	3	15.8%	6	18.2%	7	22.6%
Wet	13	20.0%	5	35.7%	2	18.2%	2	9.5%	4	21.1%	6	18.2%	7	22.6%
Menstruation†														
Bathing	35	52.2%	12	75.0%	7	58.3%	13	61.9%	3	16.7%	19	51.4%	16	53.3%
Washing cloth	34	50.7%	8	50.0%	8	66.7%	9	42.9%	9	50.0%	19	51.4%	16	53.3%
Drying cloth	31	46.3%	10	62.5%	3	25.0%	11	52.4%	7	38.9%	16	43.2%	15	50.0%
General discomfort	29	43.3%	11	68.8%	2	16.7%	7	33.3%	10	55.6%	12	32.4%	17	56.7%
People	25	37.3%	8	50.0%	4	33.3%	7	33.3%	6	33.3%	13	35.1%	12	40.0%
Pain	23	34.3%	8	50.0%	4	33.3%	7	33.3%	6	33.3%	13	35.1%	12	40.0%
Feel dirty	20	29.9%	8	50.0%	4	33.3%	3	14.3%	5	27.8%	9	24.3%	11	36.7%
Restrictions	20	29.9%	7	43.8%	3	25.0%	6	28.6%	4	22.2%	13	35.1%	7	23.3%
Irregularity	18	26.9%	4	25.0%	6	50.0%	6	28.6%	2	11.1%	14	37.8%	4	13.3%
Need support	15	22.4%	4	25.0%	2	16.7%	7	33.3%	2	11.1%	10	27.0%	5	16.7%

*Only concerns listed by ≥20% of participants overall in table. See supplement for all concerns.

†Six women did not indicate urination concerns, four did not indicate defecation concerns, two did not indicate menstruation concerns.

**Figure 1:**
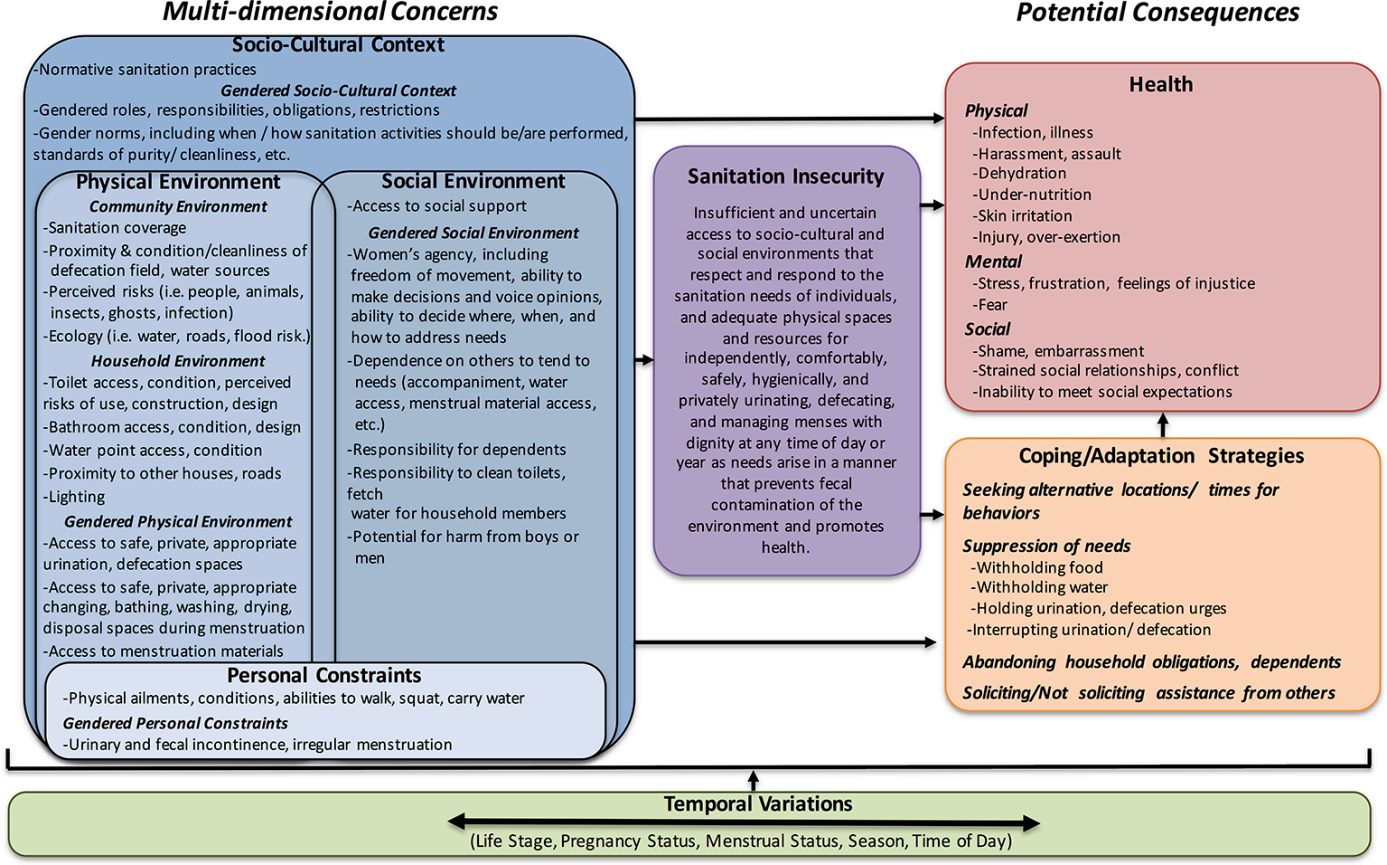
Sanitation insecurity is defined by varied and multidimensional concerns, which can lead to various health outcomes and adaptation and coping strategies. Temporal variations can influence concerns, sanitation insecurity, outcomes, and coping and adaptation strategies.

Based on the concerns noted, the conceptual model (figure 1) depicts how sanitation insecurity is defined by concerns across multiple dimensions and can lead to various health consequences and strategies for adapting or coping. The dimensions are nested and include: the sociocultural context; the physical environment; the social environment and personal constraints. Physical environment and social environment are overlapping; concerns from one dimension are largely inter-related with those of the other. Furthermore, we highlight how gender is a prominent element of each dimension. The model also shows how life course stage, season, time of day and menstrual status influence concerns, sanitation insecurity and potential consequences. In the following sections, we present thematic results from interviews and FGDs by dimension to elucidate the concerns reported and potential impacts on behaviour and health. We then discuss the resulting definition of sanitation insecurity.

### Sociocultural context

Several of the urination, defecation and menstruation concerns women described focused on upholding their gendered roles within the household and community. Women were expected to defecate at dawn or dusk for discretion, and defecation or urination could not interfere with responsibilities, like cooking or caring for family members. Men, they reported, could go as needed. Some worried about not having urges at appropriate times, or having urges at inappropriate times, particularly if ill. While one toilet owner and several non-owners said that toilet ownership enabled use as needed, some women in large households said toilets were often occupied by others.

Women, most often RMWs, were concerned about their needs disrupting household work and feared the consequences of abandoning obligations or dependents:

Once I had gone out to urinate leaving my kids at home. I told my daughter to watch her baby brother until I returned. My mother-in-law was angry as how I could leave behind two small kids… So there is always a fear if we leave behind kids. (FLI, RMW, Toilet)

Most women adapted by suppressing:

Say it is time for children to go to school. No matter what, [I] will have to make them ready by 9:30…if you have the pressure to urinate or defecate, you have to suppress. (FGD, MW, Toilet)

Women also worried about the expectation to help dependents with insecurity, outcomes, andinsecurity, outcomes, andtheir needs:

When [our mother-in-law] is going to urinate … we are forced to take (her)… If we do not go, she will urinate on the bed…When she gets pressure she will ask us to go with her to the field and if we do not accompany, she defecates on the bed. (FGD, MW, Toilet)

Some women thought having a toilet would facilitate helping dependents, but others said toilets created additional work, like fetching water or cleaning. Supporting others was particularly difficult when pregnant.

Menstruation interfered with the responsibilities women were expected to fulfil. Women said menstruation made them ‘polluted’, restricted activities and caused frustration, confusion, embarrassment and a sense of injustice:

(Menstruation) may start on some religious occasion then I can’t perform any rituals…. Boys don’t have this problem…we are the ones who suffer…there is chance of huge public embarrassment. (FLI, UMW, Toilet)

Menstruating women can only fulfil obligations like cooking or childcare *after* bathing:

I go to the pond, bring a bucket of water and come back…But until then I don’t do any other works. Even if the child cries, I won’t pacify him. This is the problem at that time. (FLI, OW, No Toilet)

Overall, women lacked control, many saying ‘What to do?’ after describing concerns. Some exercised control by limiting food and water:

I do not eat at night out of the fear that I will have the pressure to defecate… Recently I had been admitted in the hospital as I reduced eating. The doctor was angry… he said that if you do not eat at night you will die. (FGD, UMW, Toilet)

#### Physical environment

Women in all stages worried most about *where* they urinated, defecated, bathed when menstruation started, and washed, dried and disposed of their menstrual materials. Women expressed these concerns regardless of whether they owned a toilet (see table 2).

Three-quarters of women worried about where they urinated. While most participants who owned toilets reported having used them to defecate (FLI: 95%; FGD: 100%), far fewer participants reported ever having used them to urinate (FLI: 14%; FGD 41%). Women considered toilets unfit for urination:

We have to change our clothes if we go to the toilet. We do not enter the house in the same clothes we were wearing during defecation. We do not go to urinate in the toilet. (FLI, MW, Toilet)

Women typically urinated in their backyards, worrying about privacy and filth. They feared acquiring a ‘urine infection’ from urinating where someone had previously gone: "People have fear of urine infection… Say if a diabetic is sitting to urinate…If we go and urinate there, that disease will come to our body" (FGD, MW, No Toilet). This concern was most common among UMW (40%), RMW (36%) and MW (29%), who worried infertility or harm to unborn children may result. Many women wanted a private, enclosed space for urinating.

Almost all women without toilets expressed concerns about where they defecated (97%). Women were specifically worried about defecation sites lacking privacy or being filthy: "The field is dirty… There will be defecation over defecation, how to sit above it?" (FLI, OW, No Toilet). These conditions led women to worry about health, particularly women without toilets (toilet: 15%; no toilet 26%): "In the field, all defecate… If you sit to defecate on it, you get many types of diseases. That fear is there" (FLI, UMW, No Toilet). Women in FGDs discussed how they were even more concerned about where they urinated and defecated during menstruation because they perceived an increased infection risk: "Then the bleeding that we have is direct so it has direct connection with the body… so feel scared. There is more worry for infection" (FGD, UMW, No Toilet).

Women coped with open defecation environments that were filthy with faeces, urine and mud, or that lacked privacy by walking far to alternative locations or by waiting for privacy. Women also waited if men or boys occupied areas where they would bathe after defecating. However, waiting or walking led women to worry about being gone too long, interfering with household obligations. Time spent and obligations missed were of greatest concern to UMW and MW:

At times we face trouble… If a boy is coming to the river after defecation for bathing…then we have to wait until he finishes and goes. (FLI, UMW, Toilet)

Although fewer women with toilets expressed concern about where they defecated, 52% of toilet owners indicated that where they defecated was concerning to them. Toilets were not always perceived to be better than fields. One RMW stopped using the toilet when pregnant due to perceived risks:

When I was pregnant with my son, I mostly did not go to the latrine…I used to go out in the open. I would not have seen who had urinated (in the latrine) and whether they washed or not…if their diseases infect us then our child would be affected. (FLI, RMW, Toilet)

RMW were more concerned than others that their toilets were unusable because of broken doors or roofs, visible faeces or odour. Without doors, women worried about privacy; without roofs, women worried about weather. Some RMW said they were forced to use toilets, and missed ‘roaming’ with friends to defecate. More women with toilets (36%) expressed worry about accessing water for defecation than those without (19%). Toilets lacked direct water access, so users had to bring water for flushing and cleaning themselves. Women who defecated outside typically went near water sources to facilitate cleaning. Water fetching for defecation was a deterrent to toilet use, particularly among elderly, infirm or pregnant women: "We have become old. To fetch a bucket of water is difficult" (FGH, OW, Toilet).

Women with and without toilets worried about urinating or defecating in the monsoon season. They felt conditions were dirtier due to mud and standing water, resulting in more work cleaning clothing, greater risks to health due to exposure, increased fear of injury from slipping and greater challenges finding suitable locations. Women with functional, roofed toilets indicated that they used them for defecation during the monsoons, even if they usually practised open defecation: "In the rainy season we don’t go outside… We have to walk on the mud and slush, hold an umbrella and go and still get wet. So I don’t go out, use the latrine instead" (FLI, UMW, Toilet). Furthermore, not all toilets could be used year-round because of construction quality or flooding. Women adapted:

We have to walk in water, which is up to the chest level… The toilet will be filled with water, so we cannot use it…Our father-in-law ties a huge piece of wood in between two coconut trees for we daughter-in-laws to defecate. (FLI, MW, Toilet)

Navigating terrain at night during monsoons was very challenging. Advanced age, illness or pregnancy augmented difficulty. Women worried that all locations were unsafe at night because of darkness, distance and potential harm from men, animals, insects and ‘ghosts’:

At night in our jungles there are many animals… have that fear something would bite. (FLI, UMW, No Toilet)

Here in our neighbouring village… Three or four (men) lifted the girl and raped her…she was lying almost dead… Be it daughter-in-law or daughters, where will they go? We have no fear. We are 60, 65, 70, we think who will rape us? (FGD, OW, Toilet)

During menstruation, women also worried about where they were to manage their needs and were specifically concerned about lacking private, convenient and clean spaces for bathing when menstruation started, changing and disposing materials and washing and drying reusable menstrual cloths or soiled clothing. These activities necessitate discretion and often require access to several different spaces, whether ponds, water pumps or rooms in the home. They thought toilets were unsuitable locations for changing or washing materials because they lacked water, or were considered unhealthy: "If we wash it in the toilet where we go to defecate and urinate, we will have disease" (FLI, RMW, Toilet). Only one woman mentioned occasionally bathing inside and only three mentioned ever washing menstrual cloths inside: "Nobody gets to see. I close the door from that side. They would think ‘daughter has gone to defecate’. Meanwhile, I wash the cloth" (FLI, UMW, Toilet).

Women worried that accessing water sources for bathing and washing cloths, clothing and bedding (if menstruation begins when sleeping) was not always feasible. These activities were typically carried out at the water source, which were not always private. Water access was of greatest concern to RMW, who had restricted mobility and largely depended on others for water or had to use sources that were nearby and unclean or not always private.

Several women, particularly UMW and RMW, wanted disposable pads but indicated that distance to vendors or an inability to go to markets themselves limited their access. Pads were preferred because cloth was difficult to wash and dry, made women feel dirty or could cause leaks or chaffing. One UMW described the many difficulties she faced managing menstruation:

We go to the pond and wash. Those who have tube well, they fetch water … and wash in the backyard … I have hatred because it is difficult to wash the cloth. We are not able to use sanitary pads. As the market is a little far away, we will get them only when we go ourselves … Will we ask men and boys to get it for us? …We have to use cloth and feel dirty to wash. (FLI, UMW, Toilet)

Disposal of used materials was primarily a concern for RMW, who used commercial pads more than others, had restricted mobility and were less familiar with their community surroundings. They discussed throwing pads in ponds, rivers and forested areas:

I only use (pads)…If I throw it (outside), they will know it is mine. I will feel bad. If there was a toilet, would have put it there… Here, I throw in the jungle. (FLI, MW, No Toilet)

Seasonal conditions and nightfall exacerbated menstruation concerns. Women worried about managing menstruation during monsoon or winter months because of the wet or cold weather, which was even harder if menstruation started at night. Washing menstrual cloth during the rains was challenging, but drying was harder. Cloths could blow off lines, becoming dirty again or would not fully dry and be worn while damp, potentially leading to chaffed skin.

#### Social environment

Women worried about being seen and shamed when urinating, defecating, entering or leaving toilets, bathing at menstrual onset or washing, drying and disposing of materials. UMW were most concerned about being shamed and hurting their reputations and marriage prospects. If they had privacy, women worried it was ephemeral:

When we defecate outside and suddenly any male comes over, we stand up. We either hold the feces at that moment or if it was already out as we stood up then we get it all over our legs. (FGD, MW, No Toilet)

Regarding menstruation, one UMW said:

If we wash at day time, there would be people moving around… people will look at us and will say that girl has no brains… we need a place where if we wash the cloth no one can see. (FLI, UMW, No Toilet)

Regardless of toilet ownership, women were concerned about needing social support for all activities, particularly women with no or few female family members: "If we would be cooking…will have to look for someone to watch… Will have to rush" (FLI, MW, No Toilet). Women worried most about finding support at night, when they feared harm. Without support, women adapted by using suboptimal locations, suppressing or going alone:

(My husband) must be thinking that he is working all day and my wife is disturbing me and saying ‘come let’s go defecate’. So once I thought I will not wake him up …I was sitting there to defecate, it must be 2am… someone clapped. I was scared… He said ‘You did not call me! How did you go alone!' (FLI, MW, Toilet)

When menstruation starts, women had difficulty getting support for bathing, particularly during monsoons or at night:

Tension is—who will I call to go along with me? I can’t touch the clothes I have to change into. Suppose it starts at midnight. People are already sleeping deeply. Will they wake up when I call them? … You will surely feel guilty or not? (FLI, MW, Toilet)

#### Personal constraints

Many women noted concerns that related to their own physical issues or abilities to tend to their needs. For example, walking to distant defecation locations and squatting to urinate or defecate were worrisome for older and pregnant women, causing exhaustion from substantial physical exertion and apprehension about falling, particularly when navigating unsteady terrain during inhospitable weather. Some OW who previously preferred open defecation expressed interest in toilets so they could walk less. OW who already had toilets, however, indicated that fetching water to clean and flush toilets was straining. A few women reported concerns and frustration regarding urinary and faecal incontinence or irregular menstruation:

Whenever I want to defecate urgently, then my hand and legs get soiled with it. (FLI, MW, Toilet)

### No urination, defecation and menstruation concerns

Four interview participants had no urination concerns (one MW, three OW), two had no defecation concerns (one UMW, one MW), two had neither urination nor defecation concerns (one UMW, one RMW) and two had no menstruation concerns (one MW, one OW). Women with no urination concerns had drains in their courtyards for night use or areas that enabled privacy for daytime use (ie, bathroom, shed, secluded backyard); two indicated that they had lights so felt safe at night and two comfortably used umbrellas. Those with no concerns about defecation had roofed toilets that were usable year-round.

### Sanitation insecurity definition

Drawing on these findings, we propose a definition for *sanitation insecurity* that accounts for the multidimensional concerns reported by women and the factors that influence those concerns:

Insufficient and uncertain access to a socio-cultural and social environments that respect and respond to the sanitation needs of individuals, and to adequate physical spaces and resources for independently, comfortably, safely, hygienically, and privately urinating, defecating, and managing menses with dignity at any time of day or year as needs arise in a manner that prevents fecal contamination of the environment and promotes health.

This definition integrates the gendered, sociocultural context (respects, responds, independently; as needs arise, ie, not dependent or suppressing for gendered responsibilities), physical environment (insufficient and uncertain access; adequate spaces/resources; comfort; cleanliness; safety), the social environment (privacy; dignity), personal needs (urination; defecation; menstruation) and temporal variability (any time, as needs arise). It also necessarily contains reference to health and the need to prevent environmental faecal contamination. The terms intentionally remain broad to enable applicability across the population given varied needs. Adequate spaces may include both toilets and bathing areas to enable urination and menstrual management; resources may include water access, vessels for hauling water, shoes, lights/torches, menstrual management materials and social support among others.

## Discussion

Our research elucidated a definition of sanitation insecurity from women’s voiced defecation, urination and menstruation concerns. This research is among a growing number of sanitation studies including women at various life stages,^17 18^ but uniquely includes older women. Our findings revealed that women have many shared concerns, but that intensity varies by life stage, time of day, season and toilet ownership. These concerns demonstrate that, as hypothesised, sanitation insecurity is multidimensional, extending beyond facility access. Our definition acknowledges that the sociocultural context, physical environment, social environment and personal constraints collectively influence women’s sanitation experiences and each dimension includes gender as a key element.

The Millennium Development Goal target to increase ‘improved sanitation’ coverage may have limited how sanitation programming was conceived of and actualised by focusing exclusively on toilet construction. The Sustainable Development Goals specifically prioritise the needs of women and girls.^4^ Efforts to enable women and girls to address their needs require transformative approaches that also address the gendered, sociocultural context that strains women despite facility access.

Providing sanitation has been framed as a human right, fundamental for dignity and privacy.^40^ To address this right, however, *users* must consider facilities private and dignified for all needs. The Swachh Bharat Mission in India is focusing on building toilets, but toilets alone do not address women’s needs, which may explain suboptimal use and an overall stated preference for open defecation.^6 10 36 41^ In this study, many women with toilets perceived minimal benefit over open defecation; they lacked direct water access for postdefecation cleaning and flushing, roofs or doors for shelter and privacy, were too dark for night use and were not necessarily cleaner or more comfortable and convenient than outdoor spaces. Research has suggested that women may only become more vulnerable as the construction of pour-flush toilets increases and water becomes scarce.^42^ Currently, toilet ownership enables a choice, not a solution. For toilets to become part of the solution, women need to be engaged in decision making about their facilities. The government of India should encourage the non-governmental organisations that are promoting toilets as part of the Swachh Bharat Mission to actively involve women. Current research reports that these organisations ‘came and asked for men’, not women.^21^

Other researchers have questioned the appropriateness of sanitation facilities for women.^35 43^ Consistent with research that found toilets only to be used for defection,^44^ few women in our study used toilets to urinate or manage menstruation; women considered toilets unsuitable for these needs. Globally, menstrual hygiene is gaining recognition as a public health issue,^45^ but remains ‘largely absent from the sanitation vocabulary’.^46^ Women’s experiences of urination and associated health risks are understudied, likely because urination has not been linked to infectious diseases. Still, perceived health risks—regardless of whether or not they are actual risks—and lack of privacy or resources for menstruation and urination activities were prominent concerns, particularly for unmarried and recently married women. These experiences cause stress and assaults to dignity and status due to public exposure, as reported by others.^16 17 19^ Toilet availability does not necessarily enable the privacy women need. Rather, as in a study from Rajasthan, toilets can serve to expose women’s menstrual status by removing their freedom to privately dispose of materials away from the home.^47^ Women in our study overwhelmingly requested enclosed spaces for private urination and menstrual management. While creating facilities conducive to all of women’s needs may be costly, continuing to invest in under-used facilities is an expensive endeavour. Given that the Swachh Bharat Mission has no prescribed sanitation model, the government of India could promote the construction of facilities that include bathing spaces or other gender-responsive elements.

Women have reported open defecation to be pleasurable, convenient, comfortable, healthy and to enable socialising outside the home.^36 41^ Women in our study also discussed socialising during open defecation, a practice many unmarried women worried about leaving behind on marriage. These findings show that toilets may take away social freedoms, underscoring how sanitation is more than a facility, but an array of behaviours and needs influenced by broader norms that are currently overlooked by large-scale sanitation programmes.

The WASH (water, sanitation and hygiene) sector typically focuses on changing the physical environment to improve sanitation, however women had concerns beyond this dimension. The WASH sector can change the physical environment to mitigate concerns related to other dimensions. To address social environment concerns, women should decide where to place toilets to optimise accessibility and safety; low-cost lights could be installed for safer, independent use and locks could enable privacy. To address personal constraints, water could be available within toilets to eliminate fetching, walkways could be constructed to prevent falling and elevated seats or rails could aid the elderly, infirm and pregnant. Furthermore, sanitation programming should include messaging that aims to ameliorate the sanitation constraints women face because of their gender. Women are expected to sideline their sanitation needs for household obligations and the needs of others (see also Khanna and Das^20^). Sanitation programmes are missing opportunities to empower women by not tackling these issues outright.

### Strengths and limitations

The inclusion of various methodologies enhanced the validity of findings. Employing interviews and FGDs enabled data triangulation; following interviews with FGDs enabled validation of initial interview conclusions; including women of different life stages and varied latrine access enabled comparison and identification of exceptional cases and free-listing permitted quantification of concerns, increasing the validity of generalisations about the population.^48^ We did not engage men, children or urban residents, which potentially limited the findings. While we did not have recently married women represented in FGDs, we do have their voices represented through interviews. Given that FGD findings served to verify and support interview findings, we do not believe their lack of participation impacted results. We also did not have representatives of all caste categories in each life stage. Mixed-caste FGDs may have impacted what individuals felt comfortable communicating. Further research should investigate how applicable our sanitation insecurity definition is to other populations, including men and children, and other settings—like urban areas, schools, public spaces—to discern if the findings herein are specific to rural women in Odisha or are widely applicable. Exploring other facets of sanitation beyond defecation, urination and menstruation, like waste management, or facets of other dimension, like policy, could elucidate additional insights.

## Conclusion

This research revealed that women at different life stages in rural Odisha, India have a multitude of unaddressed urination, defecation and menstruation concerns, and informed a definition and conceptual model for sanitation insecurity. Ideally, our findings will encourage further research in other settings with other populations to validate or refine the definition and model. This research has inspired the subsequent development of a measure^29^ that can be used to assess sanitation insecurity and evaluate whether programmes influence sanitation insecurity scores and, in turn, influence other outcomes like psychosocial distress or gender inequity among others.
