# Supplementary material for: Understanding and defining sanitation insecurity: women’s gendered experiences of urination, defecation and menstruation in rural Odisha, India
Source: BMJ Glob Health. 2017 Oct 9;2(4):e000414. doi: 10.1136/bmjgh-2017-000414 (PMC5640070; doi:10.1136/bmjgh-2017-000414)
Supplement: Supplementary file 1 [file bmjgh-2017-000414supp001.pdf]

Supplemental Table 1a: Concerns noted by participants in free list activities and definitions used for coding

Concern	*Definition*
Themes in all Urination, Defecation and Menstruation Lists	
**Fear**	Fear when going to urinate/ defecate/ manage menses, whether of animals, ghosts, people, or harsh weather.
**Feel Dirty**	Concern about feeling or getting dirty when urinating/ defecating/ managing menses.
**Health**	Concern that health is compromised when urinating/ defecating or in relation to menstruation.
**Need Support**	Concern unable to urinate/ defecate/ manage menses alone because of need for company or support.
**People**	Concern of being seen by people when urinating/ defecating/ managing menses or that menstrual materials will be seen.
**Shame**	Concern of being publically shamed or feeling ashamed if seen by others when urinating/ defecating/ managing menses.
**Support Barrier**	Concern that someone will not provide support / will get upset if asked to accompany to urinate/ defecate/ manage menses.
**Water**	Concern getting and carrying water for urinating/ defecating/ managing menses is a great difficulty.
**Work**	Concern that workload related to urination/ defecation/ menstrual management activities is high.
Themes in Urination and Defecation Lists	
**Dependents**	Concern for well-being of dependents - child, elderly, infirm- when leaving them to urinate/ defecate.
**Difficulty-Pain**	Concern about pain or difficulty related to urination/ defecation, including general body pain, strain, etc.
**Fall**	Concern for falling when going to or in the act of urinating/ defecating.
**Harm**	Concern about being harmed when urinating/ defecating, whether by an animal, person, or environmental conditions.
**Incontinence**	Concern about the inability to hold urination/ defecation due to physical ailment.
**Light**	Concern about not having light when urinating/ defecating at night.
**Menstruation**	Concern about increased difficulty urinating/ defecating at the time of menstruation.
**No Proper Facility**	Concern that there is not a proper facility for urinating/ defecating available.
**Place**	Concern the place available and used for urinating/ defecating is problematic, whether dirty, far, hard to access, in the open, etc.
**Squat**	Concern about having difficulty sitting, squatting, or getting up and down when urinating/ defecating.
**Stand**	Concern for need to stand up in the middle of urination/ defecation if someone suddenly appears.
**Support Others**	Concern that there is difficulty providing support to others for their urination/ defecation needs.
**Suppress**	Concern about the need to suppress because various factors prevent attention to personal needs.
**Urgency**	Concern about experiencing and sudden, strong urge to urinate/ defecate.
**Walk**	Concern about walking to the place for urination/ defecation.
**Wet**	Concern that she will have to get wet when going to urinate/defecate during the rains.

Supplemental Table 1b: Concerns noted by participants in free list activities and definitions used for coding *(Continued)*

Concern	*Definition*
Themes in Urination List Only	
**Infection**	Concern for infection if unintentionally urinating over the urine of others.
**Limit Water**	Concern about the need to limit water in order to reduce need for urination.
**Urine Contact**	Concern about accidentally touching urine and therefore becoming untouchable or making others untouchable.
**Urine On Self**	Concern about getting urine on the body because of inability to suppress.
Themes in Defecation List Only	
**Cleaning Self**	Concern about personal cleaning after defecation.
**Concern For Others**	Concerns for others' safety or needs when defecating, typically their concern for daughters or daughters-in-law.
**Defecate On Self**	Concern about getting feces on the body because of inability to suppress.
**Defecation Time**	Concerns about the amount of time defecation takes to complete because location is far, others around, cleaning extensive, etc.
**Fixed Time**	Concern about having to defecate at a fixed time of day.
**Forced Latrine Use**	Concern about being forced to use the latrine against will, either all the time or during certain times of day or year.
**Future Needs**	Concerns about defecating in the future given deteriorating health, inevitable departure of daughters to marriage, etc.
**Latrine Conditions**	Concern that the conditions of the household latrine are poor.
**Latrine Unusable**	Concern that the latrine available is unsuitable for defecating.
**Limit Food**	Concern about the need to limit food in order to reduce need for defecation.
**No Money**	Concern for lacking money to construct or maintain a latrine.
**No Gov. Support**	Concern for insufficient support, financial or otherwise, from government to build or maintain a latrine.
**Obligations**	Concern regarding the need to attend to household obligations or responsibilities to others before tending to defecation needs.
**Scold**	Concern about being scolded due to defecation behavior (i.e. taking time, going to wrong place, improperly caring for others).

Supplemental Table 1c: Concerns noted by participants in free list activities and definitions used for coding *(Continued)*

Concern	*Definition*
Themes in Menstruation List Only	
**Access to Materials**	Concern about barriers to getting materials wanted /needed.
**Bathing**	Concern about bathing at onset of menstruation, regardless of time of day, night, season.
**Changing Cloth**	Concern with changing the cloth or pad, whether finding a place, not having privacy, etc.
**Disposal**	Concern about where and how to dispose of menstrual cloths or pads.
**Drying Cloth**	Concerns about where/ how to dry cloth after washing.
**Falling Cloth**	Concern that cloth will fall from clothing.
**Fertility**	Concern about fertility related to menstrual cycle, whether about ability to have children or plan for them due to irregularity.
**Forced Separation**	Concern about the need to 'stay separate' at night until bathing possible due to menstrual onset.
**General Discomfort**	Concern about generally feeling uncomfortable, bad, weak, or irritated because of menstruation.
**Heavy Bleeding**	Concern about heavy menstrual flow.
**Household Duties**	Concern about ability to effectively perform household work during menstruation.
**Irregularity**	Concern about not having a regular menstrual cycle.
**Leaks**	Concern for menstrual leaks on cloths, bed, etc.
**Mobility**	Concern about inability to move freely during menstruation, because of cloth, pad, restrictions, discomfort, etc.
**Odor**	Concern that body, cloth or clothes is generating a bad odor.
**Pain**	Concern about pain, whether in the head, stomach, legs, back or hands.
**Restrictions**	Concern for restrictions or requirements that are imposed because menstruation.
**Sleep**	Concern about inability to sleep well during menstruation.
**Start when Away**	Concern that menstrual cycle will start when away from home.
**Storing Cloth**	Concern about storing the reusable menstrual cloth between uses.
**Untouchability**	Concern about being untouchable, making things untouchable, or others touching things made untouchable by menstruation.
**Urination**	Concern with urinating during menstruation.
**Washing Cloth**	Concern with washing cloths used to absorb menstrual blood.
**Wounds**	Concern about getting wounds on legs from their menstrual pads or cloths.

Supplemental Table 2: Type and frequency of urination-related concerns overall, and by participant type and latrine status

Concern	All		1. Unmarried (UM)		2. Recently Married (RM)		3. Married (M)		4. Older Woman (OW)		Latrine At Home		No Latrine At Home	
	N=63		n=15		n=11		n=21		n=16		n=34		n=29	
**Place**	47	74.6%	11	73.3%	10	90.9%	14	66.7%	12	75.0%	25	73.5%	22	75.9%
**People**	42	66.7%	14	93.3%	6	54.5%	12	57.1%	10	62.5%	22	64.7%	20	69.0%
**Fear**	40	63.5%	13	86.7%	10	90.9%	10	47.6%	7	43.8%	22	64.7%	17	58.6%
**Need Support**	26	41.3%	10	66.7%	5	45.5%	7	33.3%	4	25.0%	14	41.2%	12	41.4%
**Wet**	21	33.3%	9	60.0%	3	27.3%	4	19.0%	5	31.3%	9	26.5%	12	41.4%
**Squat**	21	33.3%	0	0.0%	3	27.3%	6	28.6%	12	75.0%	13	38.2%	8	27.6%
**Urine Infection**	19	30.2%	6	40.0%	4	36.4%	6	28.6%	3	18.8%	10	29.4%	9	31.0%
**Get Dirty**	17	27.0%	9	60.0%	3	27.3%	4	19.0%	1	6.3%	8	23.5%	9	31.0%
**Suppress**	16	25.4%	5	33.3%	5	45.5%	3	14.3%	3	18.8%	11	32.4%	5	17.2%
**Work**	12	19.0%	5	33.3%	1	9.1%	4	19.0%	2	12.5%	4	11.8%	8	27.6%
**No Proper Facility**	11	17.5%	4	26.7%	3	27.3%	1	4.8%	3	18.8%	5	14.7%	6	20.7%
**Shame**	10	15.9%	6	40.0%	0	0.0%	3	14.3%	1	6.3%	6	17.6%	4	13.8%
**Support Others**	9	14.3%	3	20.0%	0	0.0%	3	14.3%	3	18.8%	5	14.7%	4	13.8%
**Walk**	9	14.3%	0	0.0%	1	9.1%	3	14.3%	5	31.3%	3	8.8%	6	20.7%
**Support Barrier**	8	12.7%	5	33.3%	0	0.0%	3	14.3%	0	0.0%	2	5.9%	6	20.7%
**Stand**	8	12.7%	5	33.3%	1	9.1%	1	4.8%	1	6.3%	4	11.8%	4	13.8%
**Urine On Self**	8	12.7%	3	20.0%	1	9.1%	2	9.5%	2	12.5%	3	8.8%	5	17.2%
**Dependents**	7	11.1%	2	13.3%	2	18.2%	2	9.5%	1	6.3%	4	11.8%	3	10.3%
**Urine Contact**	6	9.5%	1	6.7%	0	0.0%	3	14.3%	2	12.5%	1	2.9%	5	17.2%
**Harm**	5	7.9%	1	6.7%	0	0.0%	1	4.8%	3	18.8%	1	2.9%	4	13.8%
**Urgency**	4	6.3%	0	0.0%	0	0.0%	2	9.5%	2	12.5%	1	2.9%	3	10.3%
**Incontinence**	4	6.3%	0	0.0%	0	0.0%	2	9.5%	2	12.5%	2	5.9%	2	6.9%
**Menstruation**	4	6.3%	2	13.3%	0	0.0%	1	4.8%	1	6.3%	3	8.8%	1	3.4%
**Fall**	4	6.3%	1	6.7%	2	18.2%	0	0.0%	1	6.3%	2	5.9%	2	6.9%
**Health**	4	6.3%	2	13.3%	0	0.0%	0	0.0%	2	12.5%	1	2.9%	3	10.3%
**Difficulty-Pain**	4	6.3%	0	0.0%	1	9.1%	2	9.5%	1	6.3%	4	11.8%	0	0.0%
**Water**	3	4.8%	0	0.0%	2	18.2%	0	0.0%	1	6.3%	2	5.9%	1	3.4%

Only one respondent mentioned each of the following concerns: Limit Water (RM); Light (RM).
Six women did not indicate any concerns related to urination: 1 UM, 1 RM, 1 M, and 3 OW; 3 have latrines and 3 do not.

Supplemental Table 3: Type and frequency of defecation-related concerns overall, and by participant type and latrine status

Concern	All		1. Unmarried (UM)		2. Recently Married (RM)		3. Married (M)		4. Older Woman (OW)		Latrine At Home		No Latrine At Home	
	N=65		n=14		n=11		n=21		n=19		n=33		n=31	
**Place**	47	72.3%	10	71.4%	8	72.7%	15	71.4%	14	73.7%	17	51.5%	30	96.8%
**Fear**	36	55.4%	10	71.4%	5	45.5%	12	57.1%	9	47.4%	16	48.5%	20	64.5%
**Need Support**	33	50.8%	5	35.7%	6	54.5%	13	61.9%	9	47.4%	16	48.5%	17	54.8%
**People**	27	41.5%	9	64.3%	5	45.5%	7	33.3%	6	31.6%	8	24.2%	19	61.3%
**No Proper Facility**	23	35.4%	3	21.4%	3	27.3%	10	47.6%	7	36.8%	1	3.0%	22	71.0%
**Get Dirty**	21	32.3%	5	35.7%	5	45.5%	6	28.6%	5	26.3%	6	18.2%	15	48.4%
**Support Others**	20	30.8%	3	21.4%	3	27.3%	4	19.0%	10	52.6%	9	27.3%	11	35.5%
**Water**	18	27.7%	3	21.4%	3	27.3%	7	33.3%	4	21.1%	12	36.4%	6	19.4%
**Walk**	17	26.2%	1	7.1%	1	9.1%	5	23.8%	9	47.4%	6	18.2%	11	35.5%
**Suppress**	15	23.1%	6	42.9%	2	18.2%	5	23.8%	2	10.5%	6	18.2%	9	29.0%
**Dependents**	14	21.5%	2	14.3%	3	27.3%	5	23.8%	4	21.1%	8	24.2%	6	19.4%
**Health**	13	20.0%	5	35.7%	1	9.1%	4	19.0%	3	15.8%	5	15.2%	8	25.8%
**Squat**	13	20.0%	0	0.0%	2	18.2%	6	28.6%	5	26.3%	8	24.2%	5	16.1%
**Support Barrier**	13	20.0%	4	28.6%	2	18.2%	4	19.0%	3	15.8%	6	18.2%	7	22.6%
**Wet**	13	20.0%	5	35.7%	2	18.2%	2	9.5%	4	21.1%	6	18.2%	7	22.6%
**Shame**	12	18.5%	6	42.9%	1	9.1%	3	14.3%	2	10.5%	4	12.1%	8	25.8%
**Latrine Conditions**	11	16.9%	1	7.1%	5	45.5%	3	14.3%	2	10.5%	10	30.3%	1	3.2%
**Obligations**	11	16.9%	4	28.6%	0	0.0%	6	28.6%	1	5.3%	5	15.2%	6	19.4%
**Urgency**	10	15.4%	3	21.4%	0	0.0%	4	19.0%	3	15.8%	2	6.1%	8	25.8%
**Latrine Unusable**	10	15.4%	1	7.1%	3	27.3%	3	14.3%	3	15.8%	10	30.3%	0	0.0%
**Work**	9	13.8%	0	0.0%	1	9.1%	5	23.8%	3	15.8%	4	12.1%	5	16.1%
**Harm**	8	12.3%	2	14.3%	0	0.0%	5	23.8%	1	5.3%	1	3.0%	7	22.6%
**Fall**	7	10.8%	0	0.0%	1	9.1%	2	9.5%	4	21.1%	1	3.0%	6	19.4%
**Stand**	7	10.8%	2	14.3%	2	18.2%	0	0.0%	3	15.8%	0	0.0%	7	22.6%
**Defecation Time**	6	9.2%	2	14.3%	1	9.1%	3	14.3%	0	0.0%	3	9.1%	3	9.7%
**Difficulty-Pain**	6	9.2%	0	0.0%	0	0.0%	3	14.3%	3	15.8%	3	9.1%	3	9.7%
**No Money**	5	7.7%	0	0.0%	1	9.1%	3	14.3%	1	5.3%	1	3.0%	4	12.9%
**Scold**	5	7.7%	1	7.1%	0	0.0%	3	14.3%	1	5.3%	0	0.0%	5	16.1%
**Cleaning Self**	4	6.2%	0	0.0%	0	0.0%	2	9.5%	2	10.5%	2	6.1%	2	6.5%
**Forced Latrine Use**	4	6.2%	1	7.1%	3	27.3%	0	0.0%	0	0.0%	4	12.1%	0	0.0%
**Defecate On Self**	3	4.6%	1	7.1%	0	0.0%	2	9.5%	0	0.0%	1	3.0%	2	6.5%
**Limit Food**	3	4.6%	0	0.0%	0	0.0%	3	14.3%	0	0.0%	0	0.0%	3	9.7%

Only two respondents mentioned each of the following concerns: Concern For Others (1 M, 1 OW); Fixed Time (2 RM); Light (2 RM); Future Needs (1 M, 1 OW).
Only one respondent mentioned each of the following concerns: No Support (M); Incontinence (M); Menstruation (OW).
Four women did not indicate any concerns related to defecation 2 UM, 1 RM, and 1 M; All 4 have latrines.

Supplemental Table 4: Type and frequency of menstruation-related concerns overall, by participant type, and latrine status

Concern	All N=67		1. Unmarried (UM) n=16		2. Recently Married (RM) n=12		3. Married (M) n=21		4. Older Woman (OW) n=18		Latrine At Home n=37		No Latrine At Home n=30	
**Bathing**	35	52.2%	12	75.0%	7	58.3%	13	61.9%	3	16.7%	19	51.4%	16	53.3%
**Washing Cloth**	34	50.7%	8	50.0%	8	66.7%	9	42.9%	9	50.0%	19	51.4%	16	53.3%
**Drying Cloth**	31	46.3%	10	62.5%	3	25.0%	11	52.4%	7	38.9%	16	43.2%	15	50.0%
**General Discomfort**	29	43.3%	11	68.8%	2	16.7%	7	33.3%	10	55.6%	12	32.4%	17	56.7%
**People**	25	37.3%	8	50.0%	4	33.3%	7	33.3%	6	33.3%	13	35.1%	12	40.0%
**Pain**	23	34.3%	8	50.0%	4	33.3%	7	33.3%	6	33.3%	13	35.1%	12	40.0%
**Feel Dirty**	20	29.9%	8	50.0%	4	33.3%	3	14.3%	5	27.8%	9	24.3%	11	36.7%
**Restrictions**	20	29.9%	7	43.8%	3	25.0%	6	28.6%	4	22.2%	13	35.1%	7	23.3%
**Irregularity**	18	26.9%	4	25.0%	6	50.0%	6	28.6%	2	11.1%	14	37.8%	4	13.3%
**Need Support**	15	22.4%	4	25.0%	2	16.7%	7	33.3%	2	11.1%	10	27.0%	5	16.7%
**Work**	13	19.4%	1	6.3%	3	25.0%	5	23.8%	4	22.2%	4	10.8%	9	30.0%
**Wounds**	13	19.4%	3	18.8%	2	16.7%	5	23.8%	3	16.7%	6	16.2%	7	23.3%
**Leaks**	12	17.9%	3	18.8%	2	16.7%	4	19.0%	3	16.7%	7	18.9%	5	16.7%
**Obligations**	12	17.9%	2	12.5%	1	8.3%	4	19.0%	5	27.8%	4	10.8%	8	26.7%
**Water**	12	17.9%	4	25.0%	4	33.3%	2	9.5%	2	11.1%	6	16.2%	6	20.0%
**Forced Separation**	11	16.4%	2	12.5%	2	16.7%	4	19.0%	3	16.7%	5	13.5%	6	20.0%
**Access to Materials**	10	14.9%	5	31.3%	4	33.3%	1	4.8%	0	0.0%	8	21.6%	2	6.7%
**Shame**	8	11.9%	2	12.5%	2	16.7%	3	14.3%	1	5.6%	5	13.5%	3	10.0%
**Constrained Mobility**	7	10.4%	1	6.3%	4	33.3%	2	9.5%	0	0.0%	4	10.8%	3	10.0%
**Heavy Bleeding**	7	10.4%	1	6.3%	0	0.0%	3	14.3%	3	16.7%	2	5.4%	5	16.7%
**Health**	6	9.0%	1	6.3%	3	25.0%	1	4.8%	1	5.6%	5	13.5%	1	3.3%
**Odor**	6	9.0%	2	12.5%	0	0.0%	2	9.5%	2	11.1%	2	5.4%	4	13.3%
**Disposal**	5	7.5%	1	6.3%	3	25.0%	1	4.8%	0	0.0%	4	10.8%	1	3.3%
**Untouchability**	5	7.5%	1	6.3%	1	8.3%	2	9.5%	1	5.6%	2	5.4%	3	10.0%
**Changing Cloth**	4	6.0%	2	12.5%	2	16.7%	0	0.0%	0	0.0%	3	8.1%	1	3.3%
**Sleep**	4	6.0%	0	0.0%	0	0.0%	3	14.3%	1	5.6%	2	5.4%	2	6.7%
**Support Barrier**	4	6.0%	2	12.5%	0	0.0%	2	9.5%	0	0.0%	4	10.8%	0	0.0%

Only three respondents mentioned each of the following concerns: Fear (2 UM, 1 RM); Fertility (1 RM, 2 M); Storing Cloth (1 UM, 1 RM, 1 OW).
Only two respondents mentioned each of the following concerns: Falling Cloth (1 UM, 1 RM); Start when Away (1 UM, 1 M); Urination (1 UM, 1 M).
Two women did not indicate any concerns related to menstruation: 1 M and 1 OW; neither have latrines.
